# Toward Understanding the Catalytic Mechanism of Human Paraoxonase 1: Site-Specific Mutagenesis at Position 192

**DOI:** 10.1371/journal.pone.0147999

**Published:** 2016-02-01

**Authors:** Geetika Aggarwal, Rameshwar Prajapati, Rajan K. Tripathy, Priyanka Bajaj, A. R. Satvik Iyengar, Abhay T. Sangamwar, Abhay H. Pande

**Affiliations:** 1 Department of Biotechnology, National Institute of Pharmaceutical Education and Research (NIPER), Sector 67, S.A.S. Nagar (Mohali) -160062, Punjab, India; 2 Department of Pharmacoinformatics, National Institute of Pharmaceutical Education and Research (NIPER), Sector 67, S.A.S. Nagar (Mohali) -160062, Punjab, India; University of Toulouse - Laboratoire d'Ingénierie des Systèmes Biologiques et des Procédés, FRANCE

## Abstract

Human paraoxonase 1 (h-PON1) is a serum enzyme that can hydrolyze a variety of substrates. The enzyme exhibits anti-inflammatory, anti-oxidative, anti-atherogenic, anti-diabetic, anti-microbial and organophosphate-hydrolyzing activities. Thus, h-PON1 is a strong candidate for the development of therapeutic intervention against a variety conditions in human. However, the crystal structure of h-PON1 is not solved and the molecular details of how the enzyme hydrolyzes different substrates are not clear yet. Understanding the catalytic mechanism(s) of h-PON1 is important in developing the enzyme for therapeutic use. Literature suggests that R/Q polymorphism at position 192 in h-PON1 dramatically modulates the substrate specificity of the enzyme. In order to understand the role of the amino acid residue at position 192 of h-PON1 in its various hydrolytic activities, site-specific mutagenesis at position 192 was done in this study. The mutant enzymes were produced using *Escherichia coli* expression system and their hydrolytic activities were compared against a panel of substrates. Molecular dynamics simulation studies were employed on selected recombinant h-PON1 (rh-PON1) mutants to understand the effect of amino acid substitutions at position 192 on the structural features of the active site of the enzyme. Our results suggest that, depending on the type of substrate, presence of a particular amino acid residue at position 192 differentially alters the micro-environment of the active site of the enzyme resulting in the engagement of different subsets of amino acid residues in the binding and the processing of substrates. The result advances our understanding of the catalytic mechanism of h-PON1.

## Introduction

Human paraoxonase 1 (h-PON1) (EC 3.1.8.1) is a Ca^2+^-dependent enzyme with a molecular weight of ~ 45 kDa [1]. It is primarily synthesized in the liver and is secreted into the bloodstream where it is associated with a category of high density lipoprotein (HDL) particles [1–2]. The h-PON1 is a multitasking enzyme and can hydrolyze a variety of substrates (*viz*., aryl esters, thioesters, organophosphates, carbonates, lactones and thiolactones) [3–5]. Various hydrolytic activities of h-PON1 can be broadly grouped into three categories, namely aryl esterase, organophosphatase and lactonase [3–5]. Recent reports suggest that the catalytic activity of native h-PON1 is lactonase [4]. The precise physiological function(s) of h-PON1 is yet unknown, however, the enzyme has been shown to possess anti-inflammatory, anti-oxidative, anti-atherogenic, anti-diabetic, anti-microbial and OP-hydrolyzing activities [5–9]. Recently, the enzyme has also been shown to play an important role in the metabolism of certain drugs [5, 10].

The level and the activity of serum PON1 in individuals suffering from cardiovascular diseases, liver diseases, diabetes, renal diseases, cancer and obesity is considerably lower than in the normal subjects [5, 11–15]. Animals deficient in PON1 are prone to the development of these disease conditions and the over-expression of PON1 or administration of exogenously purified PON1 in these animals prevent/retard the development of these disease conditions [6–8, 16–18]. The protective role of h-PON1 in OP-poisoning is also well documented in the literature. Animals deficient in PON1 (knockout animals) are more susceptible to OP-poisoning as compared to their wild-type counterparts, and administration of exogenously purified PON1 has showed protection against OP-poisoning [5, 19–21]. Moreover, in some cases, PON1 has been shown to afford better protection than the currently available treatments of OP-poisoning [22]. Thus, h-PON1 has emerged as a strong candidate for the development of therapeutic intervention against a variety of disease conditions in humans.

The crystal structure of h-PON1 is not available yet and it is still unclear as to how the enzyme carries out different types of reactions. Recently, the crystal structures of a variant of chimeric PON1 (Chi-PON1) has been elucidated [23, 24]. Although, this variant of chi-PON1 (G2E6) shares ~ 85% sequence similarity with h-PON1 (at amino acid level), there is a considerable difference in the catalytic activities of these enzymes [25–28]. On the basis of the information gained from *in silico* analysis and enzymatic characterization of h-PON1 and Chi-PON1 different mechanisms explaining various hydrolytic activities of h-PON1 have been proposed [24, 27–28, 29–32]. It has been proposed that the His residue at position 115 (H115) plays a pivotal role in carrying out the lactonase/arylesterase activities of the enzyme, and the replacement of H with W at position 115 causes significant decrease in these activities of the enzyme [25, 28, 31, 33]. However, results from our earlier studies have shown that H115 residue of h-PON1 may not always be required for mediating the lactonase/arylesterase activities of the enzyme [27–28].

PON1 present in humans has been found to exist in two polymorphic forms; one at position 55 (L/M) and the other at position 192 (R/Q) [2–3, 5]. The catalytic activities of h-PON1 are not affected by L/M polymorphism at position 55, however, the R/Q polymorphism at position 192 modulates the OP- and lactone-hydrolyzing activities of the enzyme considerably [3–5, 20, 34–35]. However, it is not clear as to how the different amino acid residues at position 192 dramatically affect the two major activities of h-PON1. Thus, understanding the role of the amino acid residue at position 192 in the catalytic activities may help in elucidating the basic catalytic mechanism of h-PON1.

To investigate the role of position 192 in various hydrolytic activities of h-PON1, herein, we have performed site-specific mutagenesis at position 192 of h-PON1 variant containing W at position 115 (*i*.*e*., rh-PON1_(H115W,R192)_). The mutants were generated using an *E*. *coli* expression system and the enzymatic activities of the purified proteins were compared against different substrates. In an attempt to understand the effect of substitutions at position 192 on the structural features of the active site of the enzyme, MDS studies were carried out on the selected rh-PON1 mutants.

## Material and Methods

### Reagents

5-bromo-4-chloro-3`-indolyphosphate (BCIP), alkaline phosphatase-labelled anti-mouse secondary antibody, lysozyme, nitro-blue-tetrazolium (NBT) reagent, paraoxon-ethyl, protease inhibitor cocktail, urea, *m*-cresol purple, *δ*-valerolactone and tergitol NP-10 were purchased from Sigma–Aldrich, Bangalore, India. Mouse anti-PON1 primary antibody and 5-thiobutyl butyrolactone (TBBL) was a kind gift from Dr. Richard W James, University of Geneva, Switzerland and Dr. Tawfik D.S, Department of Biological Chemistry, Weizmann Institute of Science, respectively. Stratagene's Quick change site directed mutagenesis kit was purchased from Agilent Technologies, Gurgaon, India. Protein molecular weight markers and Bradford reagent were purchased from Bio-Rad, Gurgaon, India. DNA primers (oligonucleotides) used were synthesized and purchased from Sigma Aldrich, Bangalore, India. Chlorpyrifos oxon (CPO) and diisopropylfluorophosphate (DFP) were purchased from Chem. Service, Inc., West Chester, PA, USA. All other reagents used were of analytical grade. Buffers used were prepared in double distilled water.

### Site-specific mutagenesis

Construction of pET23a(+) plasmid containing a gene encoding rh-PON1_(H115W,R192)_ has been described in our previous report [28]. This plasmid was used to generate mutants of rh-PON1 in which the amino acid residue at position 192 was substituted by other amino acid residues. The mutants were generated by using Quick Change site-directed mutagenesis kit by following the procedure recommended by the manufacturer. The primers used for the introduction of the desired mutations were designed using Primer X software (www.bioinformatics.org/primerx/) and are presented in S1 Table. The mutagenized plasmids were amplified in *E*. *coli* DH5α cells, purified and the DNA sequence of the mutants was confirmed by bi-directional DNA sequencing (Eurofinn, India) [27–28]. The plasmids were then transformed into *E*. *coli* BL21(DE3) cells and the transformed cells were used for the expression and purification of the recombinant enzymes.

### Expression and purification of rh-PON1 mutants

The rh-PON1 mutants were produced by expressing the recombinant proteins as inclusion bodies (IBs) in *E*. *coli* and *in vitro* refolding of the IBs, by following the procedure described previously [36–37]. Briefly, *E*. *coli* cells containing plasmid encoding rh-PON1 mutant were grown in LB-media and the cultures were induced with 1 mM IPTG. The cells were allowed to grow further for 8 h at 37°C to over-express the recombinant proteins as IBs. The cells were then harvested by centrifugation and IBs were isolated [36–37]. IBs were then dissolved in freshly prepared 8M urea (in water) and the recombinant proteins present in the IBs were refolded to their active form by *in vitro* refolding and the active protein present in the refolding reaction mixture was purified by ion-exchange chromatography using Q-Sepharose column [36–37]. The purified proteins were analysed by SDS-PAGE and western blot analysis.

### Enzyme assays

The assays were performed in flat bottom 96-well plates at 25°C in a total volume of 200 μl using a Molecular Devices SPECTRAmax PLUS Microplate spectrophotometer [27–28]. In all assays, the appropriate blank (*i*.*e*., buffer containing the indicated substrate and no enzyme) was included to correct the spontaneous, non-enzymatic hydrolysis of the substrate. The amount of the substrate hydrolyzed (*i*.*e*. the product formed) was calculated and expressed as μMole/min/mg of the protein [27–28]. The specific activity of rh-PON1_(wt)_ was taken as 100% and the activities of the mutant proteins were then calculated with respect to the control. The hydrolysis of Pxn and *δ*-valerolactone (*δ*-val) (1 mM final concentration) was monitored by following the procedure described previously [27–28]. Hydrolysis of 5-thiobutylbutyrolactone (TBBL) (0.5 mM final concentration) was monitored using the Ellman-based colorimetric assay [38]. DFP- and CPO-hydrolyzing activity of the enzymes was measured using the acetylcholinesterase (AChE) inhibition assay, as described previously [27–28]. The final concentrations of CPO and DFP used in the assay were 75 and 200 μM, respectively [27–28]. The kinetic parameters (*K*m and *k*_cat_) were calculated for Pxn substrate by following the procedure described previously [28]. Briefly, purified rh-PON1 enzymes (0.4 μM) were separately incubated with a range of substrate concentrations (0–3 mM) and the product formation was monitored at 405 nm [28]. Kinetic parameters were obtained by the using initial velocity (*v*_0_) of the enzyme catalyzed reaction and substrate concentrations by fitting the data with Michaelis-Menten equation [28]:

$$v_{0}=k_{cat}{\left[E\right]}_{0}{\left[S\right]}_{0}/\;\left({\left[S\right]}_{0}+K_{m}\right).$$

### Homology model preparation

Homology model of rh-PON1_(wt)_ was constructed using Modeller 9v8 software package, as described previously [39–40]. Briefly, crystal structure of Chi-PON1 (G2E6 variant) was used as a template (PDB accession code 1v04). A free online software, ClustalW (www.ebi.ac.uk/Tools/msa/clustalw2) was used to the align template amino acid sequence with the sequence of rh-PON1_(wt)_ using a gap penalty of 10 and a gap extension penalty of 0.005. This alignment and the template atom files were used to generate 50 models of the rh-PON1_(wt)_ enzyme and 5 models were selected with lowest DOPE score and high mol pdf scores. These models were then energy minimized by applying spatial constrains using a free online Chimera software and the stereo-chemical quality of each model was validated by the program PROCHECK and the ERRAT plot (nihserver.mbi.ucla.edu/SAVES/). The crystal structure of Chi-PON1 contained a disordered loop region between residues 72–79, this disordered loop was constructed in each selected 5 models by using inbuilt loop refinement tool in modeller software. Finally, the model of rh-PON1_(wt)_ with the best PROCHECK and the ERRAT plot values was selected and was further utilized for the generation of the rh-PON1 mutant models. The models of rh-PON1_(H115W,R192)_, rh-PON1_(H115W,R192K)_ and rh-PON1_(H115W,R192I)_ were generated by *in silico* mutagenesis using the Pymol software [41]. The models of rh-PON1 were energy minimized using 1000 iterative cycles in Sybyl 7.1 software and were used for further study [41].

### Preparation of ligands

Ligands (phenyl acetate (Pha), Pxn, TBBL and *δ*-val) were prepared using Sybyl 7.1 software, as described previously [39–40, 42]. A short minimization (1000 iteration cycles) was performed by the Powell method to remove internal spatial strains in the molecules. The minimization was terminated when the energy gradient convergence criteria of 0.05 kcal/mol/Å was reached. The ionic state of each substrate was considered and proper charges were assigned accordingly.

### Molecular docking and Molecular dynamic simulations (MDS)

Molecular docking studies were performed by using GOLD software suite, as described previously [40]. All the studies were performed using the default GOLD fitness functions (VDW = 4.0, H-bonding = 2.5) and the default evolutionary parameters: number of operations = 100,000; population size = 100; selection pressure = 1.1; niche size = 2; migration = 10; number of islands = 5; crossover = 95 and mutation = 95 (L5). The catalytic calcium was selected as the centre of the binding site for all calculations. Fifty docking runs were performed per structure unless 3 of the 50 poses were within 1.5 Å root mean square deviation (RMSD) of each other. The outputs were exported to Silver window for the visual inspection of the binding modes and the interaction of the ligands with the amino acid residues in the active site of the protein. The best docked poses of the ligands were further subjected to MDS.

The atomic charges for the ligands were derived using the AM1-BBC method implemented in Antechamber program of the AMBER12. General amber force field (GAFF) was used to generate the missing parameters of the ligands using the Parmcheck module of the Antechamber. The AMBER99SB force field was used for the parameterization of the protein. The protein-ligand complex was then solvated in a TIP3 water box and the counter ions were added to neutralize the system. Further, the ionic strength of the system was maintained using Na^+^ and Cl^-^. The protein-ligand systems were minimized in two steps. Initially, the protein-ligand complex was restrained with a force constant of 50 kcal/mol/Å^2^ and only the solvent phase was relaxed. Then, the whole system was minimized with 500 cycles of each of the steepest descent and the conjugate gradient methods. The heating phase was performed on the NVT ensemble and the system was gradually heated to 310 K for 50 ps. The system was further equilibrated for 5 ns on NPT with a restraint force constant of 20 kcal/mol/Å^2^ on the protein-ligand complex. The final production phase was performed at 310 K and 1 atm pressure on the NPT ensemble for 50 ns. For all simulations, the step size was kept 2 fs. The SHAKE algorithm was used to constraint all the bonds containing H-atoms. The Langevin thermostat and barostat were used for the temperature and the pressure coupling, respectively. A cut off of 8 Å was taken for the non-bonded interactions and the Particle Mesh Ewald (PME) method was used to treat the long-range electrostatic interactions. VMD, Ptraj, and xmgrace were used for the trajectory analysis [39, 43].

## Results

**Fig 1:**
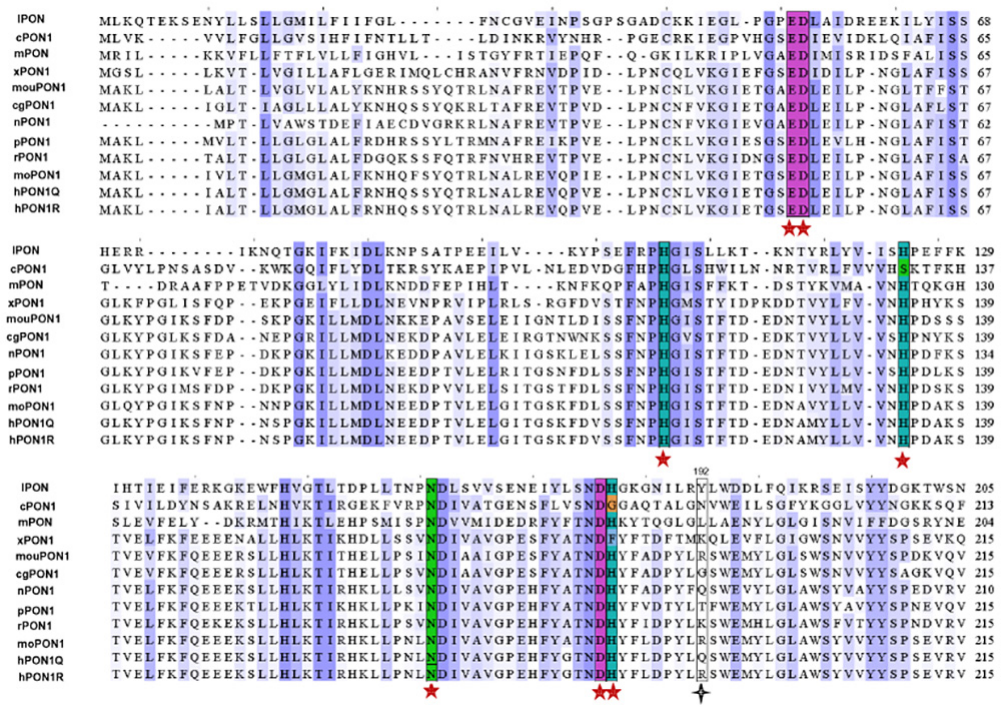
Sequence alignment of PON1 sequences from other organisms with common homology to hPON1. Alignment was performed using online ClustalW software (www.ebi.ac.uk/Tools/msa/clustalow). Abbreviations and NCBI accession codes are in parenthesis: h-PON1192Q isoform (h-PON1192Q; Accession code NP_000437.3), Oryctolagus cuniculus/rabbit (rPON1; NP_001075766.1), Sus scrofa/Pig (pPON1; NP_001090984.2), Macaca mulatta/monkey (moPON1; XP 001095992.1) Xenopus Silurana tropicalis/ Xenopus (xPON1; NP 001006848.1), Mus musculus/mouse (mouPON1; NP 035264.2), Cricetulus griseus/ Chinese hamster (CgPON1; XP_003506695) and Heterocephalus glaber/ Naked mole rat (nPON1; EHA98623.1). PON sequences from lower organisms such as Caenorhabditis elegans (cPON1; NP_491306), Maribactersp HTCC2170 (mPON1; YP_003862768) and Leptospira interrogans (lPON1; NP_710580) are aligned with human PON1 sequence. Only partial sequences of these proteins are shown. The important residues are highlighted manually using Jalview software. Sequences spanning the highly conserved residues (E53, D54, H115, H134, N168, D183 and H184 of h-PON1) shown are highlighted with different colors and marked with red asterisks. The amino acids of other sequences aligned with position 192 of h-PON1 are shown in black box and are marked with black asterisks.

Alignment of amino acid sequences of PON1 from different organisms revealed that the proposed active site amino acid residues reported in the literature [23, 33] are highly conserved, however, the residues at positions 192 (and 166) are variable (Fig 1). H-PON1 hydrolyzes a variety of substrates, however, the molecular details of how the enzyme catalyses different types of reactions are not clear yet. It was proposed that the catalytic residue H115 (and H134) mediates the lactonase and the aryl esterase activities of the enzyme [33]. However, our recent results suggest that the H115 residue is not always needed for the lactonase and aryl esterase activities of h-PON1 [27–28]. Interestingly, substitution of the amino acid residue (R/Q) at position 192 with K results in considerable retention of the lactonase activity of the enzyme, even when the H115 residue is substituted with W [27–28]. Hence, variability of amino acid residues at position 192 with respect to H115W substitution was studied to further investigate the influence of this position on various hydrolytic activities of the enzyme. In this study, we have used rh-PON1_(H115W,R192)_ variant, which contained W and R at positions 115 and 192, respectively, to generate the mutants.

### Site-specific mutagenesis

Generation and detailed characterization of rh-PON1_(H115W,R192)_, rh-PON1_(H115W,R192Q)_ and rh-PON1_(H115W,R192K)_ are described in our previous study [28]. A mutant containing C at position 192 was not generated because of the possible complications during the *in vitro* refolding of this recombinant protein. The remaining 16 mutants were generated by using Quick Change site-directed mutagenesis kit. The DNA primers used in the generation of the mutants are given in S1 Table. The presence of the desired mutation was confirmed by bi-directional DNA sequencing.

### Expression and purification of rh-PON1 mutants

Recently we have developed a procedure to produce active rh-PON1 enzymes using an *E*. *coli* expression system [36–37]. The catalytic properties of the refolded enzymes were found to be similar to their soluble counterparts [36–37]. Out of 17 mutants, two mutant proteins (i.e., rh-PON1_(H115W,R192H)_ and rh-PON1_(H115W,R192M)_) were expressed as low-molecular weight truncated products (~30 kDa) (data not shown). This could have been due to the structural changes in these mutant proteins resulting in their proteolytic degradation. The effect of mutation at position 192 on the structural stability of the recombinant proteins was also studied by using FoldX algorithm (http://foldx.crg.es/) (S2 Table) with default parameters (S1 Text). It was observed that certain mutations at position 192 (K/Q/A/I/V) stabilized the structure indicating that this position can accommodate various amino acid substitutions (S2 Table). However, in comparison to template molecule, certain mutations at position 192 (M/H) also caused increase in energy (ΔΔG < kcal/mol) and destabilized the structure. These results are consistent with the fact that rh-PON1_(H115W,R192H)_ and rh-PON1_(H115W,R192M)_ mutants were expressed as truncated products and these mutants were therefore not considered further in the study. Remaining mutants were purified by following the procedure described previously [36–37].

### Enzyme assays

The hydrolytic activities of the mutants were determined against different substrates and were compared with the activity of rh-PON1_(wt)_ (the activity of rh-PON1_(wt)_ was considered 100%).

### Comparison of OP-hydrolyzing activity

The OP-hydrolyzing activity of the rh-PON1_(wt)_ and the mutants was compared using three OP-substrates (*i*.*e*., Pxn, CPO and DFP), by following the procedures described in the Experimental section. Pxn (the active metabolite of the insecticide parathion) is the most commonly used OP-substrate for the assaying OP-hydrolyzing activity of h-PON1. CPO, a commonly used pesticide metabolite and DFP, a less hazardous structural analogue of the class-G nerve agent (NA) also served as good substrates. These three OP-substrates also differ in their structure.

**Fig 2:**
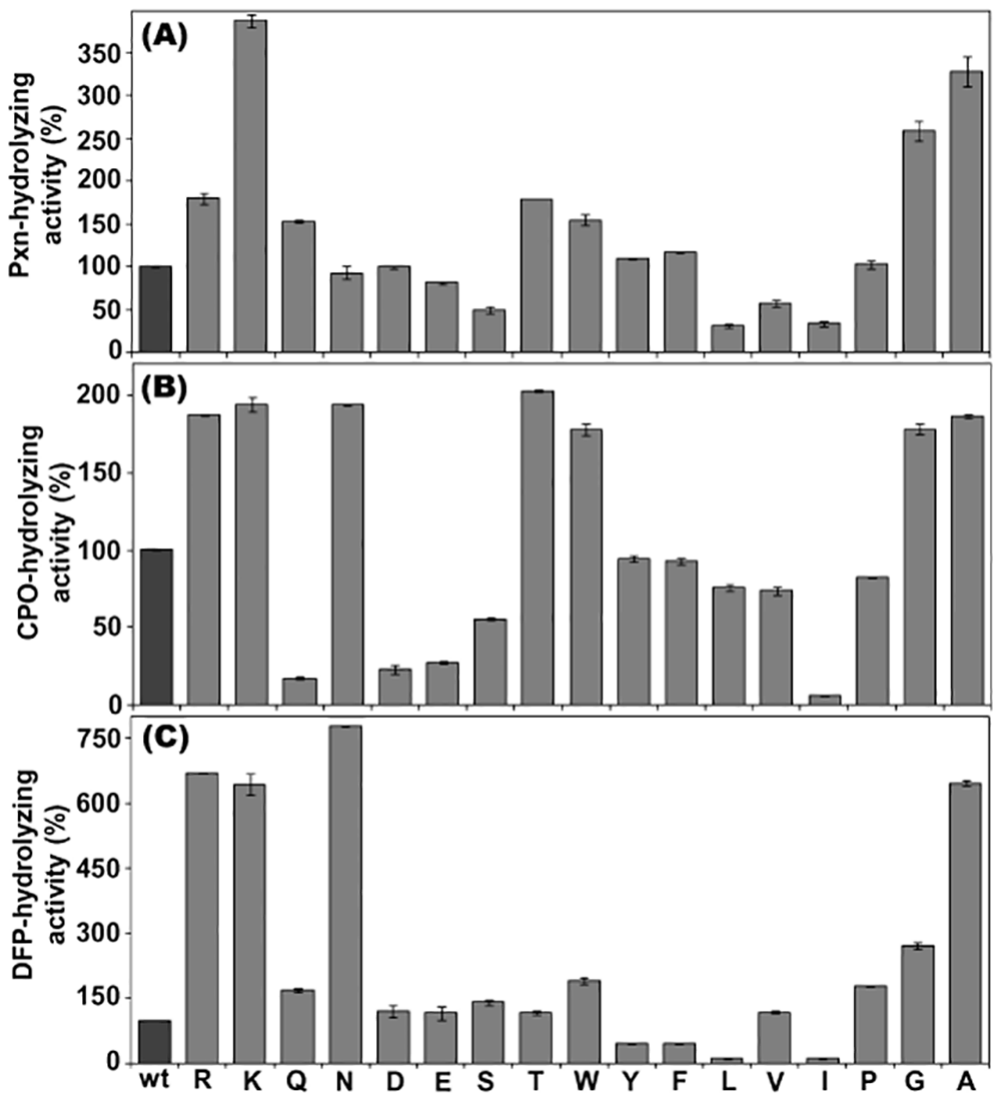
OP-hydrolyzing activity of the rh-PON1 enzymes. (A) compares the Pxn-hydrolyzing activity of the recombinant enzymes. The Pxn-hydrolyzing activity was determined using direct assay. Equal amounts of the rh-PON1 enzymes were incubated with 1 mM paraoxon in the activity buffer (50 mM Tris-HCl, pH 8.0 and containing 1 mM CaCl_2_) and the hydrolysis of Pxn was recorded at 405 nm. (B) and (C) depict the CPO- and DFP- hydrolyzing activity of the enzymes, determined by using an indirect AChE-inhibition assay, as described in the Experimental procedure. The concentration of CPO and DFP used were 75 μM and 200 μM (final concentration), respectively. The hydrolytic activity of rh-PON1_(wt)_ was taken 100% and the percentage activities of the rh-PON1 mutants were calculated. Enzymatic assays were performed in duplicate. Various mutants were named with single letter code representing the particular amino acid at position 192. **Legends:** wt, rh-PON1_(wt)_; K, rh-PON1_(H115W,R192K)_; R, rh-PON1_(H115W,R192)_; Q, rh-PON1_(H115W,R192Q);_ N, rh-PON1_(H115W,R192N)_; D, rh-PON1_(H115W,R192D)_; E, rh-PON1_(H115W,R192E);_ S, rh-PON1_(H115W,R192S);_ T, rh-PON1_(H115W,R192T)_; W, rh-PON1_(H115W,R192W)_; Y, rh-PON1_(H115W,R192Y);_ F, rh-PON1_(H115W,R192F);_ L, rh-PON1_(H115W,R192L)_; I, rh-PON1_(H115W,R192I)_; V, rh-PON1_(H115W,R192V);_ P, rh-PON1_(H115W,R192P);_ G, rh-PON1_(H115W,R192G)_; A, rh-PON1_(H115W,R192A)_.

Compared to the rh-PON1_(wt),_ most of the mutants showed an enhanced Pxn-hydrolyzing activity (Fig 2A). This was expected as substitution of H115W is known to increase the OP-hydrolyzing activity of PON1 [25, 33]. R192K substitution further increased the Pxn-hydrolyzing activity of the enzyme, while the activity of the rh-PON1_(H115W,R192Q)_ was lower than that of the rh-PON1_(H115W,R192K)_. These observations were consistent with our previous findings [28]. Substitution of N/D/E/P at position 192 resulted in the retention of the Pxn-hydrolyzing activity of the mutants to the level of rh-PON1_(wt)_, while substitution of W/Y/F resulted in only a marginal increase in the Pxn-hydrolyzing activity of the enzyme. The mutants containing L/I/V substitution at position 192 showed a considerable decrease in their Pxn-hydrolyzing activity. Interestingly, compared to rh-PON1_(wt)_, the substitution of G/A at position 192 resulted in considerable increase in the Pxn-hydrolyzing activity of the mutants (Fig 2A). Kinetic parameters for Pxn-hydrolysis were determined for a subset of rh-PON1 mutants (Table 1). *K*_*m*_ value of paraoxon towards selected rh-PON1 mutants was nearly same while *K*_*cat*_ values differs however, correlates with the Pxn-hydrolyzing activity of the mutants as in Fig 2.

**Table 1:** Kinetic parameters for hydrolysis of Pxn by rh-PON1 mutants. a,b,c- Data derived from Bajaj et al. (2014) Biochimie, 22: 210–214.

Protein	*K*_m_ (mM)	*k*_*cat*_ (s^-1^)	*k*_*cat*_/ *K*_m_ (M^-1^s^-1^) x10^3^
	Paraoxon (Pxn)		
^a^rh-PON1_(wt)_	1.2 ± 0.5	0.9 ± 0.14	0.7 ± 0.1
^b^rh-PON1_(H115W)_	0.9 ± 0.2	6.0 ± 0.10	6.6 ± 0.3
^c^rh-PON1_(H115W,R192K)_	0.9 ± 0.3	10.7 ± 0.80	10.7 ± 3.1
rh-PON1_(H115W,R192I)_	0.9 ± 0.2	0.1 ± 0.09	0.1 ± 0.1
rh-PON1_(H115W,R192S)_	1.1 ± 0.1	0.2 ± 0.22	0.2 ± 0.1
rh-PON1_(H115W,R192W)_	1.3 ± 0.1	4.2 ± 0.17	3.3 ± 0.1
rh-PON1_(H115W,R192A)_	1.2 ± 0.3	7.7 ± 2.30	6.4 ± 2.1
rh-PON1_(H115W,R192N)_	0.5 ± 0.1	3.8 ± 0.27	4.6 ± 1.3

Analysis of the CPO-hydrolyzing activity of the rh-PON1 mutants also showed a differential trend (Fig 2B). Compared to rh-PON1_(wt),_ the rh-PON1_(H115W,R192)_ and rh-PON1_(H115W,R192K)_ mutants showed an increased CPO-hydrolyzing activity, as observed earlier [25, 33]. Substitution of F/Y/L/V/G at position 192 resulted in either a complete retention or a marginal decrease in their CPO-hydrolyzing activity, while substitution of N/T/W/G/A greatly increased the CPO-hydrolyzing activity of the enzyme. The mutants containing Q/D/E/S/I substitution at position 192 showed considerable decrease in their CPO-hydrolyzing activity.

As observed with Pxn and CPO, different mutants showed differential DFP-hydrolyzing activity (Fig 2C). The mutants containing R/K/N/A substitution at position 192 showed considerable increase in their DFP-hydrolyzing activity, while D/E/S/T/V/Q/W/P/G substitution resulted in a marginal increase in the DFP-hydrolyzing activity of the enzyme. Interestingly, mutants containing Y/F/L/I substitutions at position 192 showed considerable decrease in their DFP-hydrolyzing activity.

### Comparison of lactone-hydrolyzing activity

**Fig 3:**
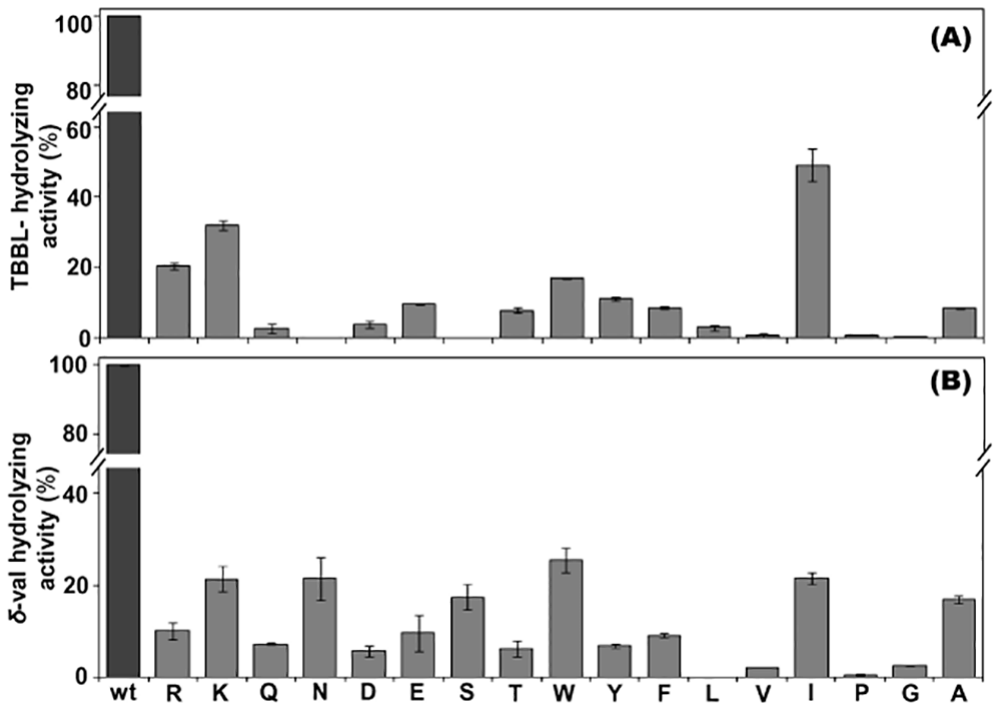
Lactone-hydrolyzing activity of the rh-PON1 enzymes. (A) and (B) compare the TBBL- and *δ*-val-hydrolyzing activity of the rh-PON1 enzymes, respectively. The TBBL-hydrolyzing activity was determined using Ellman-based colorimetric assay. Equal amounts of the rh-PON1 enzymes were separately incubated with 0.5 mM TBBL in the activity buffer containing 0.3 mM DTNB and the hydrolysis of TBBL was monitored at 412 nm. The *δ*-val-hydrolyzing activity of the rh-PON1 enzymes was determined by pH-indicator assay. The enzymes were incubated with 1 mM (in 50 mM bicine buffer pH 8.3, 1 mM CaCl_2_) and the hydrolysis of *δ*-val was monitored at 577 nm using *m*-cresol purple as the indicator. The hydrolytic activity of rh-PON1_(wt)_ was taken 100% and the percentage activities of all the rh-PON1 mutants were calculated. Enzymatic assays were performed in duplicate. Various mutants were named with single letter code representing the particular amino acid at position 192. **Legends:** same as in the legends of Fig 3.

The h-PON1 can act on a variety of lactones and lactone hydrolysis is the native activity of h-PON1 [3–4]. The effect of mutations on the lactonase activity of enzyme was compared using two structurally distinct lactones, *δ*-val and TBBL (Fig 3). Analysis of the TBBL-hydrolyzing activity showed that, compared to rh-PON1_(wt)_, substitution of R/K resulted in retention of the 20–30% of the TBBL-hydrolyzing activity of the mutants, and all other substitutions (except I) resulted in considerable decrease in the TBBL-hydrolyzing activity of the enzyme. Interestingly, compared to rh-PON1_(wt)_, substitution of I at position 192 considerably (50%) retained the TBBL-hydrolyzing activity of the enzyme (Fig 3A). Analysis of the *δ*-val-hydrolyzing activity of mutants showed that, compared to rh-PON1_(wt)_, substitution of K/I/N/W retained 20–30% of the *δ*-val-hydrolyzing activity of the enzyme and all other substitutions at position 192 resulted in considerable decrease in the activity of the mutants (Fig 3B).

The above results (Figs 2 and 3) clearly indicate that substitution of the amino acid residue at position 192 with other differentially influences the hydrolytic activity of h-PON1 and even differential effect of a particular substitution on the hydrolytic activity varies with the type (structure) of particular category of substrates (OPs and lactones).

### Homology modelling

The 3D structural model of the rh-PON1 proteins were built by using the structure of the Chi-PON1 variant (G2E6) as the template (S1A Fig). Our model contains a flexible loop region between 72–79 amino acids which are missing in the crystal structure of Chi-PON1 [23]. The 3D-fold of the generated model was verified and validated by using the program PROCHECK. Analysis of the model of the rh-PON1_(wt)_ protein revealed that the features observed in the structure of Chi-PON1 [23], *i*.*e*., the six-bladed β-propeller scaffold, the three α-helices at the top of the propeller, the putative catalytic dyad H115-H134 in the active site of the enzyme and the Ca^2+^-binding residues centred in the tunnel are well conserved in our modeled structure. The backbone dihedral distribution of all amino acid residues of the modeled structure of rh-PON1_(wt)_ was calculated by the Ramachandran plot which showed no residue in the disallowed region, 87.5% (260) residues in the most favoured region, 11.1% (33) residues in the allowed region, and 1.3% (4) residues in the generously allowed region (S1B Fig). The overall quality factor of the homology model was 84.88% (S1C Fig).

### Molecular dynamic simulations (MDS) studies

To investigate the differential effect of substitutions at position 192 on the substrate binding and catalysis, MDS studies were performed using 4 selected proteins (*i*.*e*., rh-PON1_(wt)_, rh-PON1_(H115W,R192)_, rh-PON1_(H115W,R192K)_ and rh-PON1_(H115W,R192I)_) (S1A and S2 Figs). Four ligands (Pha, Pxn, *δ*-val and TBBL) were used in our MDS studies (S3 Fig). The backbone RMSD of the rh-PON1 proteins in the protein-ligand complex during the course of MDS is given in S4 Fig. The RMSD of the rhPON1_(wt)_ was nearly similar to that of the mutant proteins indicating that the protein structures do not exhibit any asymptotic behaviour that could indicate unfolding of the protein structures (S4 Fig).

#### Interaction of the rh-PON1 proteins with aryl ester (Pha)

The RMSDs of Pha ligand in the protein-ligand complexes were monitored during the course of the MDS and were plotted as a function of time (S5A Fig). Compared to the RMSD of Pha ligand in the rh-PON1_(wt)_-Pha and the rh-PON1_(H115W,R192K)_-Pha complexes, relatively more fluctuation was observed in the rh-PON1_(H115W,R192R)_-Pha and the rh-PON1_(H115W,R192I)_-Pha complexes (S5A Fig), indicating the relative lower stability (varying degree of conformational change) of Pha in the active site of the latter. In the rh-PON1_(wt)_-Pha complex, flipping of the phenyl ring of Pha showing a π-π interaction with residues F222 as well as F292 was noted during the MDS. Contribution of the hydrophobic interactions of Pha with residues F222 or F292 in the stable binding of Pha in the active site of the h-PON1 has also been observed by others [44–45].

**Fig 4:**
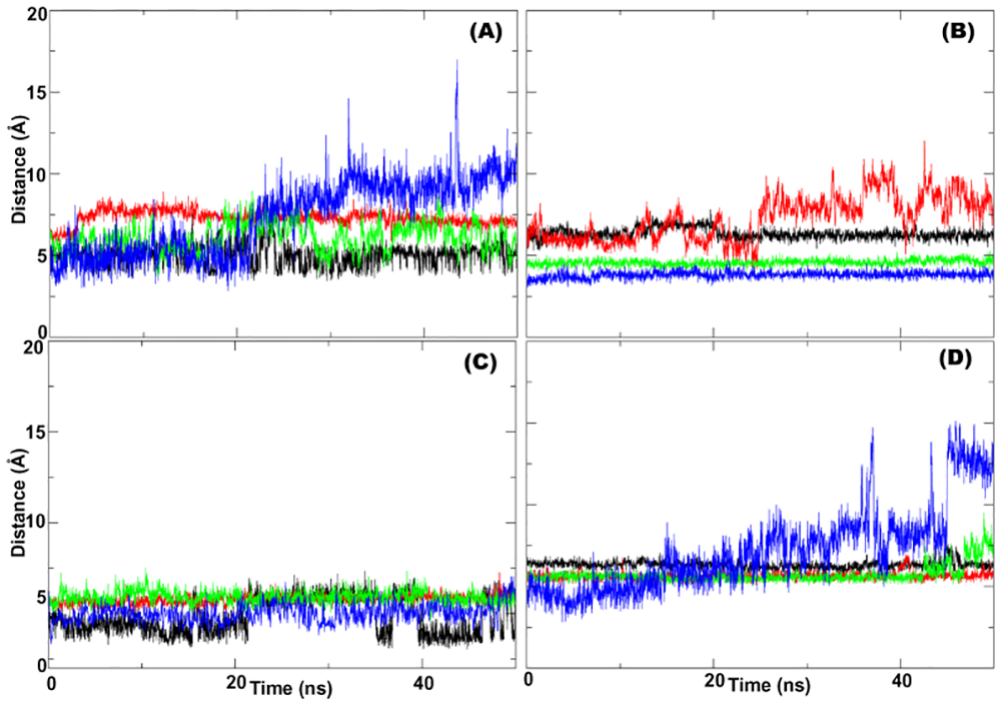
Comparison of the distance between catalytic calcium and the ligand in the protein ligand complex. The position of the ligand substrate in the active site was monitored by plotting the distance between catalytic calcium and the centre of the mass of substrate. (A-D) depict the distance between the catalytic calcium of the rh-PON1_(wt)_ (**—**), rh-PON1_(H115W,R192)_ (**—**),rh-PON1_(H115W,R192K)_ (**—**), and rh-PON1_(H115W,R192I)_ (**—**) proteins and the bound ligands over the course of the MD simulations. The ligands used were panel (A)–Pxn; panel (B)—Pha, panel (C)—*δ*-val, and panel (D)—TBBL.

Next, we analysed the distance between the catalytic calcium and the Pha ligand in the protein-ligand complexes (Fig 4). In the rh-PON1_(wt)_ and rh-PON1_(H115W,R192K)_ proteins, the Pha was found at a distance of ~ 5Å from the catalytic calcium and showed less fluctuations over the course of the MDS. In contrast, continuous fluctuation in the distance between the catalytic calcium and the bound Pha ligand was observed in the rh-PON1_(H115W,R192R)_ and rh-PON1_(H115W,R192I)_ proteins (Fig 4A). Analysis of the orientation of Pha in the protein-ligand complex revealed that in the rh-PON1_(wt),_ the oxygen atom of the carbonyl group of the Pha ligand was oriented towards H115 and the catalytic calcium. This has been observed by others too [29, 42]. On the other hand, in the rh-PON1_(H115W,R192)_-Pha and rh-PON1_(H115W,R192I)_-Pha complexes, it was noted that the oxygen atom of the carbonyl group of Pha was oriented away from W115. As a result of this the bound Pha was unable to form a stable π-π interaction either with F222 or with F292 residues in the rh-PON1_(H115W,R192)_-Pha and rh-PON1_(H115W,R192I)_-Pha complexes.

#### Interaction of the rh-PON1 proteins with OP (Pxn)

The RMSDs of the Pxn ligand in the protein-ligand complexes were plotted as a function of time (S5B Fig). The RMSD of the Pxn ligand in the rh-PON1_(wt)_-Pxn complex showed more fluctuation as compared to the RMSD of the Pxn ligand in the rh-PON1_(H115W,R192)_-Pxn and rh-PON1_(H115W,R192K)_-Pxn complexes, indicating stabilized binding of Pxn ligand in the latter complexes (S5B Fig). Interestingly, the RMSD of the Pxn ligand in the rh-PON1_(H115W,R192I)_-Pxn complex also showed considerable fluctuation, indicating a varying degree of conformational changes (and the consequent instability) in rh-PON1_(H115W,R192I)_-Pxn complex (S5B Fig).

Analysis of the distance of the catalytic calcium and bound the Pxn ligand in the protein-ligand complexes indicated that in the rh-PON1_(wt)_, the distance between the catalytic calcium and the bound Pxn was ~6 Å and was nearly constant throughout the course of the MDS (Fig 4B). In contrast, this distance was less in the rh-PON1_(H115W,R192)_ and rh-PON1_(H115W,R192K)_ mutants. In the rh-PON1_(H115W,R192I)_ a continuous increase in the distance between the catalytic calcium and the bound Pxn ligand was noted (Fig 4B). The orientation of the bound Pxn ligand in the protein-ligand complexes was also analysed during the course of the MDS. It was noted that in the rh-PON1_(wt)_, rh-PON1_(H115W,R192)_ and rh-PON1_(H115W,R192K)_ proteins, the oxygen atom of the carbonyl group of the Pxn was oriented towards D269 and the catalytic calcium throughout the MDS. These observations suggest the role of D269 residue in the binding and processing of Pxn. Previous reports also suggested the possibility of involvement of D269 residue of wild-type PON1 enzyme in the OP-hydrolyzing activity of the enzyme [29, 42].

To elucidate the effect of substitution at position 192 on the architecture of the active site, H-bonding interactions between the active site residues in the protein-ligand complexes were compared. It was observed that the H-bonding network in the rh-PON1_(H115W,R192K)_ protein was considerably different than in the rh-PON1_(wt)_ and rh-PON1_(H115W,R192)_ proteins. It was noted that the K192 residue lacked H-bonding with the oxygen atom of A137, P165 and F186 residues as compared to R192 in the rh-PON1_(wt)_ and rh-PON1_(H115W,R192)_ proteins. Also, K192 was more inclined toward the residues D183 and N166 and was able to form strong H-bonding and electrostatic interactions with the D183 and N166 residues and with the nitro group of the Pxn nitrophenyl ring. However, the R192I substitution in rh-PON1_(H115W,R192I)_ dramatically altered the H-bonding interactions between the active site residues, and no stable H-bonding interactions or electrostatic interactions between the D183 and N166 residues and the nitro group of the Pxn nitrophenyl ring was observed in this mutant.

#### Interaction of rh-PON1 proteins with lactones (δ-val and TBBL)

The interaction of the rh-PON1 proteins with lactones (*δ*-val and TBBL) was studied by the MDS study. S5 Fig shows the RMSD of *δ*-val (S5C Fig) and TBBL (S5D Fig) ligand, respectively, in the protein-ligand complexes during the course of the MDS. The RMSD of *δ*-val and TBBL in the rh-PON1_(H115W,R192)_-ligand and the rh-PON1_(H115W,R192I)_-ligand complexes showed lesser fluctuation as compared to the complexes of the rh-PON1_(wt)_ and rh-PON1_(H115W,R192K)_ proteins. This suggested a relatively more stable binding of both the lactone ligands in the active site of the former proteins. It was also noted that both the lactone ligands showed hydrophobic interactions with F222, F292, L240 and I291 residues in the rh-PON1_(wt)_ and rh-PON1_(H115W,R192I)_ proteins, while such interactions were not observed in the rh-PON1_(H115W,R192)_ and rh-PON1_(H115W,R192K)_ proteins.

Analysis of the distance of the catalytic calcium and the bound lactone ligands (Fig 4C and 4D) indicated that in rh-PON1_(wt)_ both the ligands (*δ*-val and TBBL) showed a constant distance from the catalytic calcium, while considerable fluctuation in the distances of the both ligands from the catalytic calcium was observed in the rh-PON1_(H115W,R192)_ and rh-PON1_(H115W,R192K)_ proteins. Interestingly, in rh-PON1_(H115W,R192I),_ the distance between the catalytic calcium and TBBL was also found to be constant. The analysis of the orientation of the lactone ligands in the protein-ligand complexes revealed that in rh-PON1_(wt)_, the oxygen atom of the carbonyl group of both the lactones was oriented toward the H115 residue of the protein. This observation is in agreement with the published reports [29, 42]. However, as compared to rh-PON1_(wt)_, considerably different orientation of both the lactone ligands were observed in the rh-PON1_(H115W,R192),_ rh-PON1_(H115W,R192K)_ and rh-PON1_(H115W,R192I)_ proteins. In the latter proteins the oxygen atom of the carbonyl group of the lactone was seen oriented away from the W115 residue.

**Fig 5:**
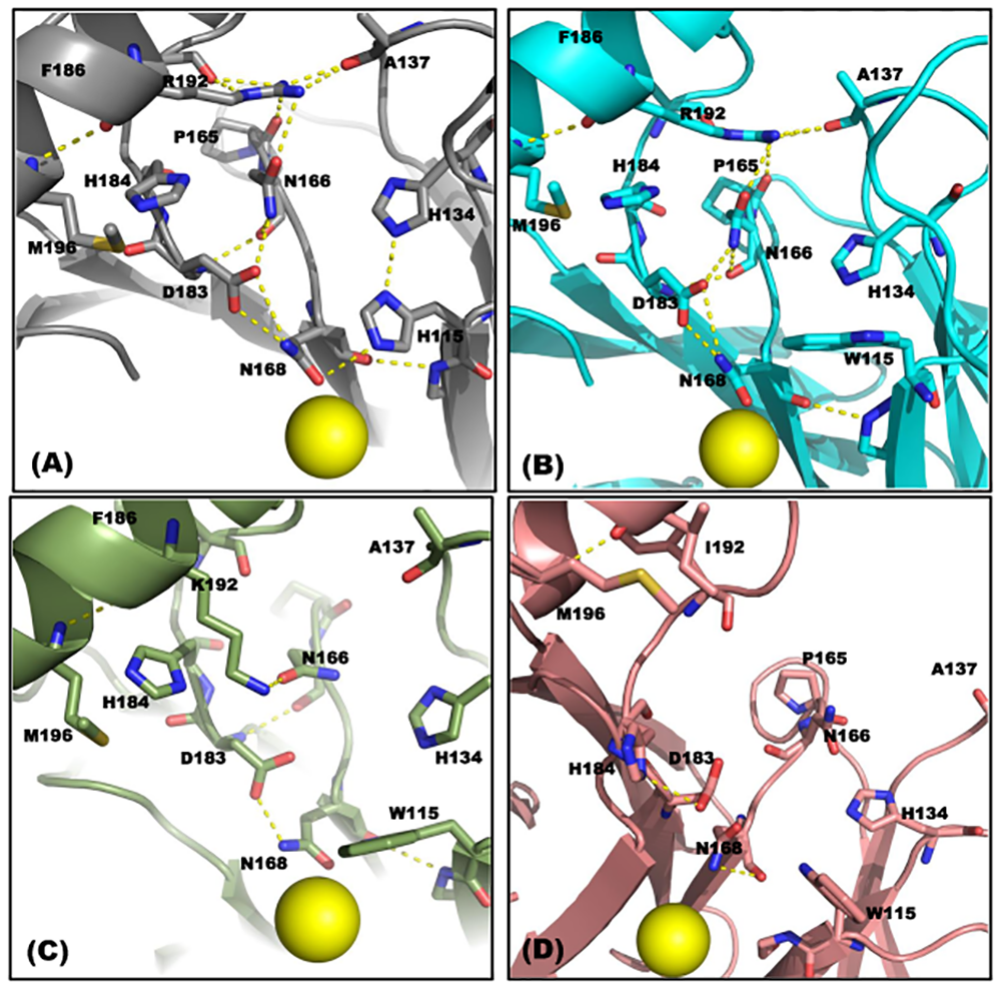
H-bonding network in the active site of the TBBL-bound rh-PON1 protein complexes. The amino acid residues of the rh-PON1 proteins are shown in stick format and colour by atom type (**red**–oxygen; **blue**–nitrogen). The yellow broken lines show H-bonding interaction between the amino acid residues. Catalytic calcium is represented by yellow spheres. **Panels (A-D)** depict rh-PON1_(wt)_, rh-PON1_(H115W,R192)_, rh-PON1_(H115W,R192K)_, and rh-PON1_(H115W,R192I)_ proteins. Differential H-bonding network around position 192 was observed in these proteins.

Analysis of the H-bonding network around position 192 in the protein-TBBL complexes (Fig 5) revealed that the network in the rh-PON1_(H115W,R192K)_ and rh-PON1_(H115W,R192I)_ proteins was considerably different from that in the rh-PON1_(wt)_ and PON1_(H115W,R192)_ proteins. In the rh-PON1_(wt)_ and rh-PON1_(H115W,R192)_ proteins, stable H-bonding interactions were observed between F186, A137, P165, N166, R192, D183 and N168 residues which were not observed in the rh-PON1_(H115W,R192K)_ and rh-PON1_(H115W,R192I)_ proteins (Fig 5). The observed difference in the H-bonding network in these proteins correlated well with the altered conformations of the amino acid residues in the active site of these proteins. For example, in the rh-PON1_(wt)_, rh-PON1_(H115W,R192)_ and rh-PON1_(H115W,R192K)_ proteins, the conformation of the H184 and D183 residues was such that no H-bonding was observed between these residues, while in the rh-PON1_(H115W,R192I)_ protein, these residues were oriented in such a way that they could form stable H-bonding interactions (Fig 5D).

**Fig 6:**
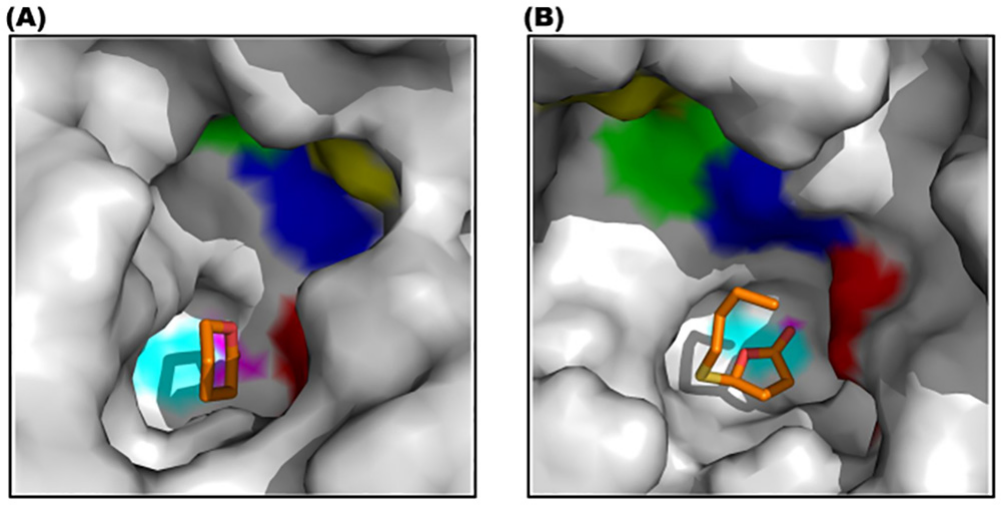
Molecular surface representation of the active site of rh-PON1_(H115W,R192I)_ protein containing *δ*-val (A) and TBBL (B) ligands. The molecular surface of the active site of the protein is shown in grey colour and the active site residues W115, D183, H184, I178, D269 and the catalytic calcium are indicated by red, blue, green, yellow, cyan and magenta colours, respectively. *δ*-val and TBBL are shown in stick model and colour by atom type (red—oxygen; yellow—sulphur; orange—carbon). Note that in the rh-PON1_(H115W,R192I)_ containing TBBL **(B)**, the oxygen atom of TBBL is oriented towards the carboxyl oxygen of D183 (blue).

Enzymatic analysis revealed that, compared to rh-PON1_(wt)_, the R192I substitution, has a differential effect on the TBBL- and *δ*-val-hydrolyzing activity (Fig 3). In order to investigate the differential effect, the orientation of both the lactone ligands in the active site of the rh-PON1_(H115W,R192I)_ protein was compared. Fig 6 shows the molecular surface representation of the active site of rh-PON1_(H115W,R192I)_ containing *δ*-val (Fig 6A) and TBBL (Fig 6B). Considerable difference in the orientation of the bound ligands was observed. The oxygen atom of the carbonyl group of TBBL was more directed towards the D183 residue with an average distance of ~5 Å (S6 Fig) while, the oxygen atom of the carbonyl group of *δ*-val was oriented away from the D183 residue (Fig 6).

## Discussion

H-PON1 can act on a variety of substrates and possesses a multitude of beneficial properties. It is a potential candidate for the development of therapeutic intervention against OP-poisoning and for a variety of disease conditions in humans [5–10]. However, how the enzyme catalyses such a variety of reactions is still unclear. Earlier it was proposed that the lactonase activity of h-PON1 is mediated by its H115/H134 dyad [20, 23, 25, 33]. However, our published results show that h-PON1 mutants containing substitutions at 115 position (W at 115 position) with K at position 192 (other than R/Q), retained considerable lactonase activity. These observations suggested that the amino acid residue(s) at position 192 plays an important role in modulating the hydrolytic activities of the h-PON1. For investigating the effect on the catalytic mechanism of h-PON1 caused due to amino acid substitutions at position 192, in this study, the site specific mutagenesis at position 192 was performed in the rh-PON1_(H115W,R192)_ variant of h-PON1. Enzymatic characterization and the MDS studies suggested that the presence of a certain amino acid residue at position 192 induces a unique change in the environment of the active (substrate-binding) site of the enzyme (*viz*, subtle change in the distance between the catalytic calcium and the substrate, orientation of the substrate in the active site, alteration in the H-bonding network around position 192 and the differential orientation of the catalytically important amino acid residue(s) in the active site, *etc*.), thereby differentially affecting the various hydrolytic activities of the enzyme. It is important to note that in the absence of the crystal structure of native h-PON1, homology models of rh-PON1 enzymes were developed by using the structure of Chi-PON1. Thus, the conclusions drawn from our *in silico* analyses could at best predict the approximate interactions taking place in the active site of h-PON1.

The effect of particular amino acid at position 192 on different activities of h-PON1 seems to be either electrostatic or structural in nature, as hypothesized recently by Ben-David et al [32]. For instance, the effect of K192 is structural on the lactonase activity (by maintaining the hydrogen bonding network leading up the active site tunnel through to helix H2), whereas it is electrostatic for Pxn-hydrolysis. That is, despite the long distance from any reacting residue, the substrate nevertheless sees the charge on K192, and this residue helps stabilizing the developing negative charge on the oxyanion [32]. Our experiments clearly support this, because insertion of positively charged residues at position 192 enhances Pxn-hydrolyzing activity of the enzyme, and insertion of negatively charged residues (D/E) depresses this activity (presumably through build-up of charge repulsion with the leaving group of substrate). Moreover, some of the other amino acid substitutions at position 192 presumably also have structural effects, because of the key involvement of position 192 in the hydrogen bonding network. In order to further validate this hypothesis, kinetic parameters for Pxn-hydrolysis were determined for a subset of rh-PON1 mutants (Table 1). The results clearly indicate that rh-PON1 mutants exhibit nearly similar binding affinity towards Pxn while possessing considerable differences in the *k*_cat_ values (Table 1). Thus, it could be inferred from these results that the mutants differ in the processing of Pxn presumably due to the electrostatic and structural effects caused by the substitutions at position 192. It seems that the orientation of Pxn in the active site of these mutants does not majorly affect its binding affinity, however, alterations in the electrostatic and structural interactions in the active site of the enzyme, caused by the mutations at position 192, considerably affect the enzyme turnover.

Literature suggests that h-PON1 shows promiscuity and its active site is capable of binding to different substrates and catalyses diverse reactions. However, the molecular basis of the enzyme promiscuity is unknown [31]. Our results also suggest that the active site of h-PON1 is highly versatile and can differentially bind and hydrolyze different substrates even of the same categories, depending on the environment of the active site. For example, on substitution of different amino acid residues at position 192, changes were observed in the conformation of the A137, F186, N168, P165, N166, H184 and D183 residues in the mutant proteins indicating the fluxional nature of these residues in the active site of the enzyme and this may contribute to a differential binding and thus hydrolysis of different substrates of the same category. For example, R192I substitution (in rh-PON1_(H115W,R192I)_) resulted in the differential hydrolysis of TBBL and *δ*-val.

Recently, it was suggested that the compartmentalization of PON1 (*viz*., binding to HDL) also differentially modulates the hydrolytic activities of enzyme [32]. It was reported that HDL-binding significantly stimulated the lactone-hydrolyzing activity of the enzyme without affecting the Pxn (OP)-hydrolyzing activity [32]. It was observed that in addition to the involvement of the first shell active site residues, certain remotely lying residues at the periphery of the active site, which form second (D183, H184) and third shell arrays (K192, N166), also participate and affect the hydrolytic activity of PON1 [32]. It was proposed that these second and third shell residues form a network of interactions which favoured the substrate binding and catalysis. Our results also support the above observations and suggest that there may exists a network of interactions involving the amino acid residue at position 192, and such a network may facilitate the alignment of the catalytic calcium and the other catalytic residues in a manner favourable for binding and processing of particular substrate(s). It appears that there exist a concerted involvement of the second and the third shell residues in governing different hydrolytic activities of h-PON1. For example, in rh-PON1_(H115W,R192I)_ mutant, conformation of the third shell residue (D183) was such that the carbonyl group of TBBL was directed towards D183 within the distance required for the possible nucleophilic attack by D183 on the TBBL. To explore this aspect, a D183S mutant (Ser is proposed to participate in the catalysis by donating a proton to the substrate (33)) was generated and its lactonase activity was determined. It was observed that D183S substitution in rh-PON1_(H115W,R192I)_, resulted in retention of lactonase activity similar to the activity of rh-PON1_(H115W,R192I)_ mutant (data not shown).

Overall, our results suggest that, due to the presence of different amino acid residues at position 192, h-PON1 can hydrolyse different substrates at different rates. In fact, a difference in the OP-hydrolyzing activity of PON1 from different species was observed recently [46]. At this juncture, we speculate that in different organisms, PON1 can participate in the metabolism of different substrates depending on the need of the organism. This aspect needs to be investigated further.

## Conclusion

It is known that amino acid residue at position 192 plays an important role in determining the substrate specificity of h-PON1 enzyme. Alignment of PON1 sequences from different organisms showing the variability at position 192 and presence of polymorphism at position 192 in natural h-PON1 indicates the importance of position 192 in the catalytic mechanism of enzyme. Mutagenesis studies at position 192 indicate that different amino acid residues at position 192 engage different subsets of amino acid residues in the active site of enzyme by forming different hydrogen-bonding network. This allows the substrates to adopt certain conformations (depending on the type of substrate) in the binding pocket of the enzyme, by either inducing electrostatic or structural effects, thereby differentially affecting various enzymatic activities of h-PON1.

## Supporting Information

S1 Fig Homology model of rh-PON1_(wt)_. (DOCX)

S2 Fig Homology model of rh-PON1_(H115W,R192)_ (A), rh-PON1_(H115W,R192K)_ (B), and rh-PON1_(H115W,R192I)_ (C) proteins. (DOCX)

S3 Fig Structures of the ligand (substrates) used in the MD simulation study. (DOCX)

S4 Fig RMSD of rh-PON1 proteins in the presence of different ligands. (DOCX)

S5 Fig RMSD of bound ligand in protein-ligand complex. (DOCX)

S6 Fig A snapshot showing binding conformation of TBBL in the active site of rh-PON1_(H115W,R192I)._ (DOCX)

S7 Fig Michaelis-Menten plot for the hydrolysis of paraoxon by the rh-PON1 enzymes. (DOCX)

S1 Table Primers used in the study. (DOCX)

S2 Table Stability analysis of rh-PON1 mutants by FoldX algorithm. (DOCX)

S1 Text Supporting methods include Sequence alignment and FoldX analysis. (DOCX)

## Abbreviations

rh-PON1: recombinant human paraoxonase 1
chi-PON1: chimeric PON1
TBBL: 5-thiobutylbutyrolactone
RMSD: root mean square deviation
MDS: molecular dynamics stimulation
IBs: inclusion bodies
AChE: acetylcholinesterase
ATCh: acetylthiocholine
CPO: chlorpyrifos oxon
NA: nerve agent
DFP: diisopropylflourophosphate
DTNB: dithionitrobenzoic acid
Pxn: paraoxon
Pha: phenyl acetate
OP: organophosphate
