# Supplementary material for: Toward Understanding the Catalytic Mechanism of Human Paraoxonase 1: Site-Specific Mutagenesis at Position 192
Source: PLoS One. 2016 Feb 1;11(2):e0147999. doi: 10.1371/journal.pone.0147999 (PMC4734699; doi:10.1371/journal.pone.0147999)
Supplement: S1 Fig — (DOCX) [file pone.0147999.s001.docx]

**Supporting information**

**
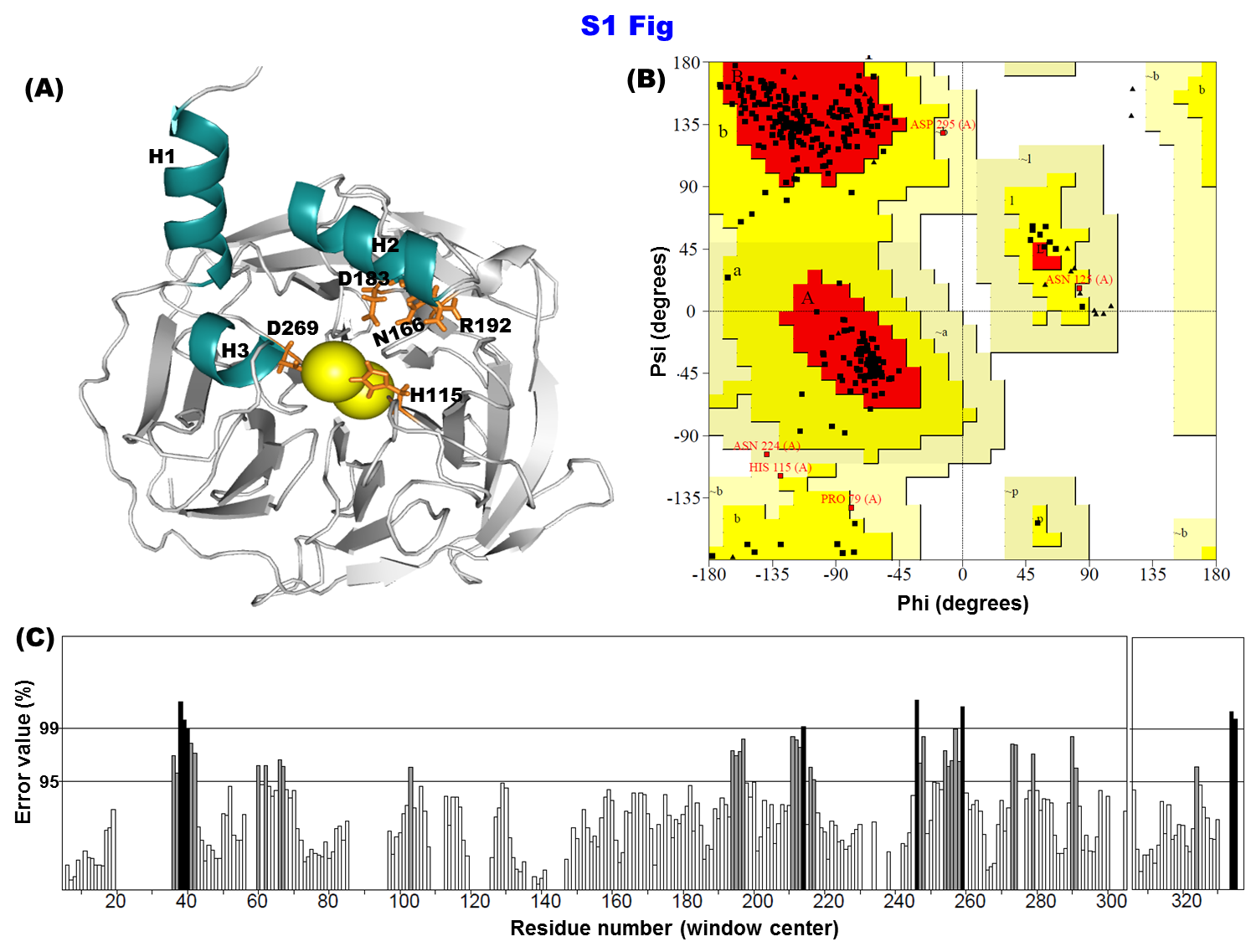
**

**S1 Fig**. **Homology model of rh-PON1_(wt)_**. **(A)** shows a ribbon diagram representing the proposed secondary structure of rh-PON1_(wt)_, viewed along the axis with the catalytic and the structural calcium (yellow spheres). The model was generated by using Modeller 9v8 software on the basis of the structure of Chi-PON1 variant, G2E6 (pdb I.D. 1V04) as a template. The amino acid residues involved in the hydrolytic activities of h-PON1 (H115, R192, N166, D183 and D269) are shown in orange stick format and the three α-helical segments of the protein (H1, H2 and H3) are shown in cyan color. **(B) and (C)** show the Ramachandran plot and the ERRAT plots, respectively of the homology model. The Ramachandran plot shows that 87.5% residues are in the most favored region with no residues in the disallowed region indicating the quality of the model in terms of the ϕ–ѱ angle. In Errat plot, the overall quality factor of homology model was 84.88 %.
