# Supplementary material for: Toward Understanding the Catalytic Mechanism of Human Paraoxonase 1: Site-Specific Mutagenesis at Position 192
Source: PLoS One. 2016 Feb 1;11(2):e0147999. doi: 10.1371/journal.pone.0147999 (PMC4734699; doi:10.1371/journal.pone.0147999)
Supplement: S2 Fig — (DOCX) [file pone.0147999.s002.docx]

**Supporting information**


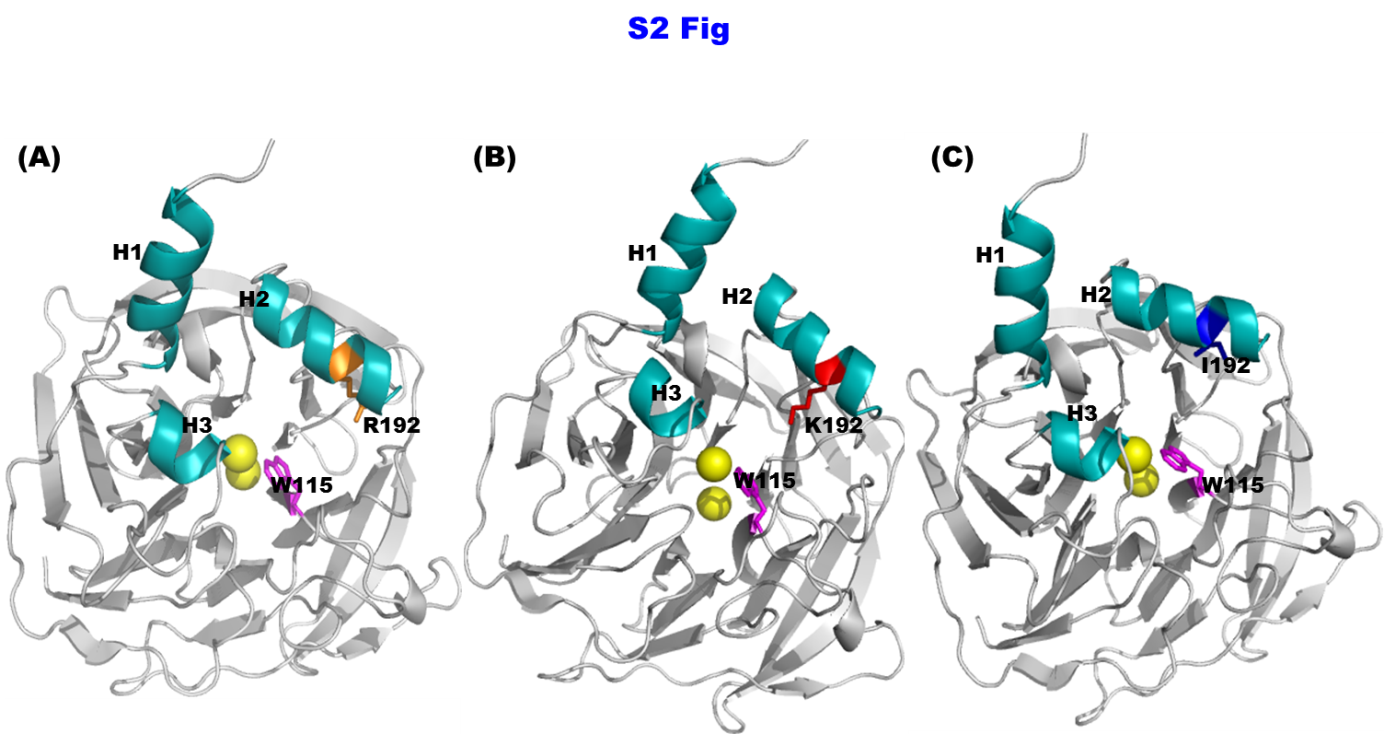


**S2 Fig**. **Homology model of rh-PON1_(H115W,R192)_ (A), rh-PON1_(H115W,R192K)_ (B), and rh-PON1_(H115W,R192I)_ (C) proteins**. The figure shows a ribbon diagram representation of the proposed secondary structures of the proteins, viewed along the axis with the catalytic and the structural calcium (yellow spheres). *In silico* mutagenesis was done on the homology model rh-PON1_(wt)_ by using mutagenesis wizard tool inbuilt in the Pymol software. The amino acid residues (mutated in this study) at positions 115 and 192 are represented in stick format and the three α-helical segments of the protein (H1, H2 and H3) are shown in cyan color. The amino acid residue W at position 115 in the three mutant models are shown in magenta color and the amino acid residue at position 192 for rh-PON1_(H115W,R192),_ rh-PON1_(H115W,R192K)_ and rh-PON1_(H115W,R192I)_ are shown in orange, red and blue color respectively.
