# Supplementary material for: Toward Understanding the Catalytic Mechanism of Human Paraoxonase 1: Site-Specific Mutagenesis at Position 192
Source: PLoS One. 2016 Feb 1;11(2):e0147999. doi: 10.1371/journal.pone.0147999 (PMC4734699; doi:10.1371/journal.pone.0147999)
Supplement: S3 Fig — (DOCX) [file pone.0147999.s003.docx]

**Supporting information**

**
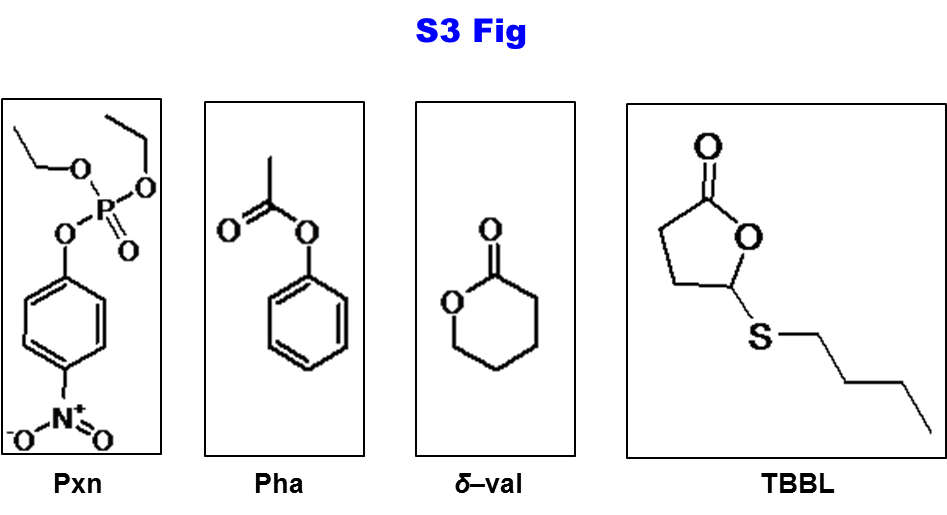
**

**S3 Fig**. **Structures of the ligand (substrates) used in the MD simulation study**.
