# Supplementary material for: Toward Understanding the Catalytic Mechanism of Human Paraoxonase 1: Site-Specific Mutagenesis at Position 192
Source: PLoS One. 2016 Feb 1;11(2):e0147999. doi: 10.1371/journal.pone.0147999 (PMC4734699; doi:10.1371/journal.pone.0147999)
Supplement: S4 Fig — (DOCX) [file pone.0147999.s004.docx]

**Supporting information**


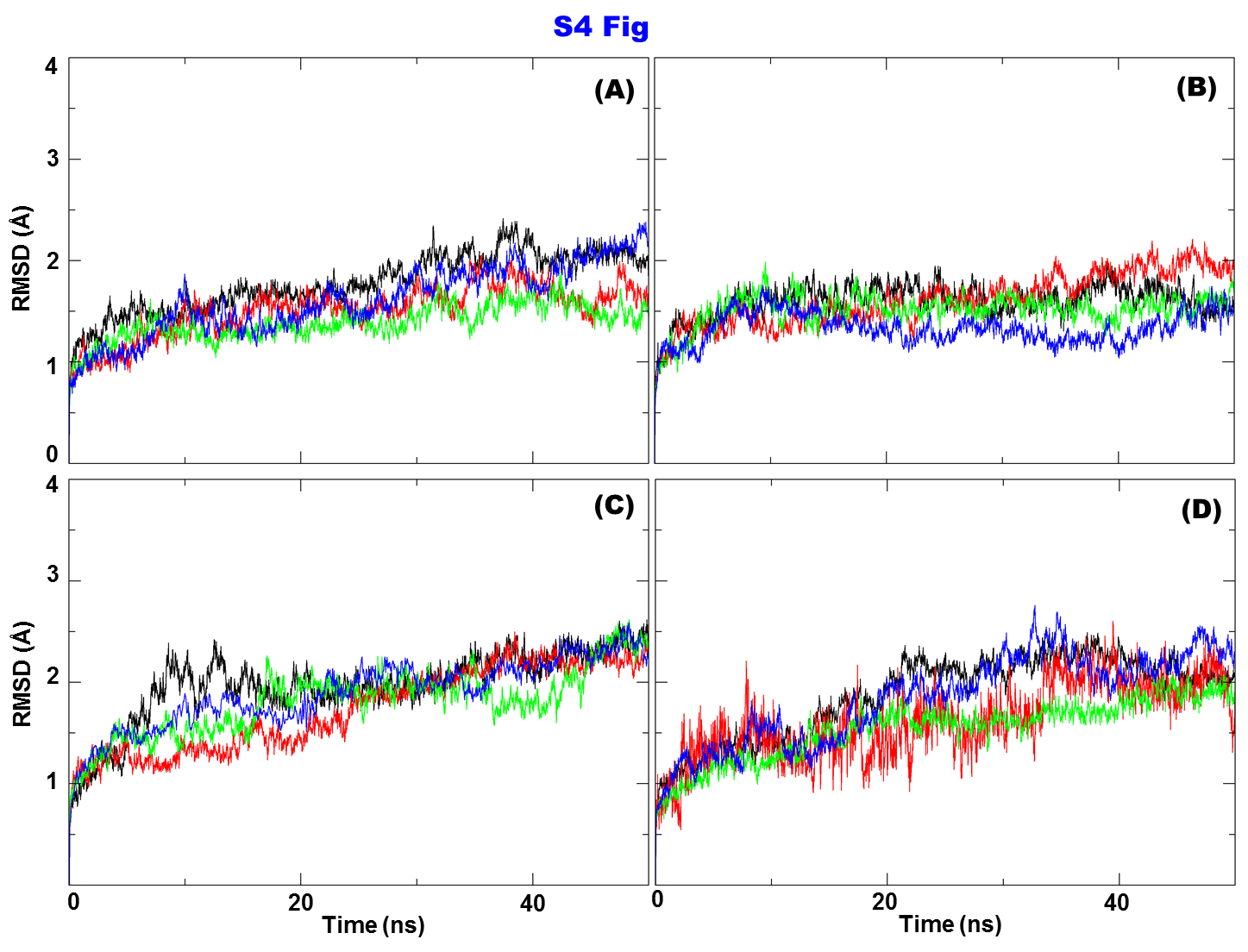


**S4 Fig**. **RMSD of rh-PON1 proteins in the presence of different ligands**. Backbone RMSD values during the course of MDS for rh-PON1_(wt)_ (**—**), rh-PON1_(H115W,R192)_ (**—**), rh-PON1_(H115W,R192K)_ (**—**), and rh-PON1_(H115W,R192I)_ (**—**) proteins were plotted with respect to the initial protein structure. The ligand substrates used were **(A)** - Pxn, **(B)** - Pha, **(C)** - *δ*-val, and **(D)** - TBBL
