# Supplementary material for: Toward Understanding the Catalytic Mechanism of Human Paraoxonase 1: Site-Specific Mutagenesis at Position 192
Source: PLoS One. 2016 Feb 1;11(2):e0147999. doi: 10.1371/journal.pone.0147999 (PMC4734699; doi:10.1371/journal.pone.0147999)
Supplement: S5 Fig — (DOCX) [file pone.0147999.s005.docx]

**Supporting information**


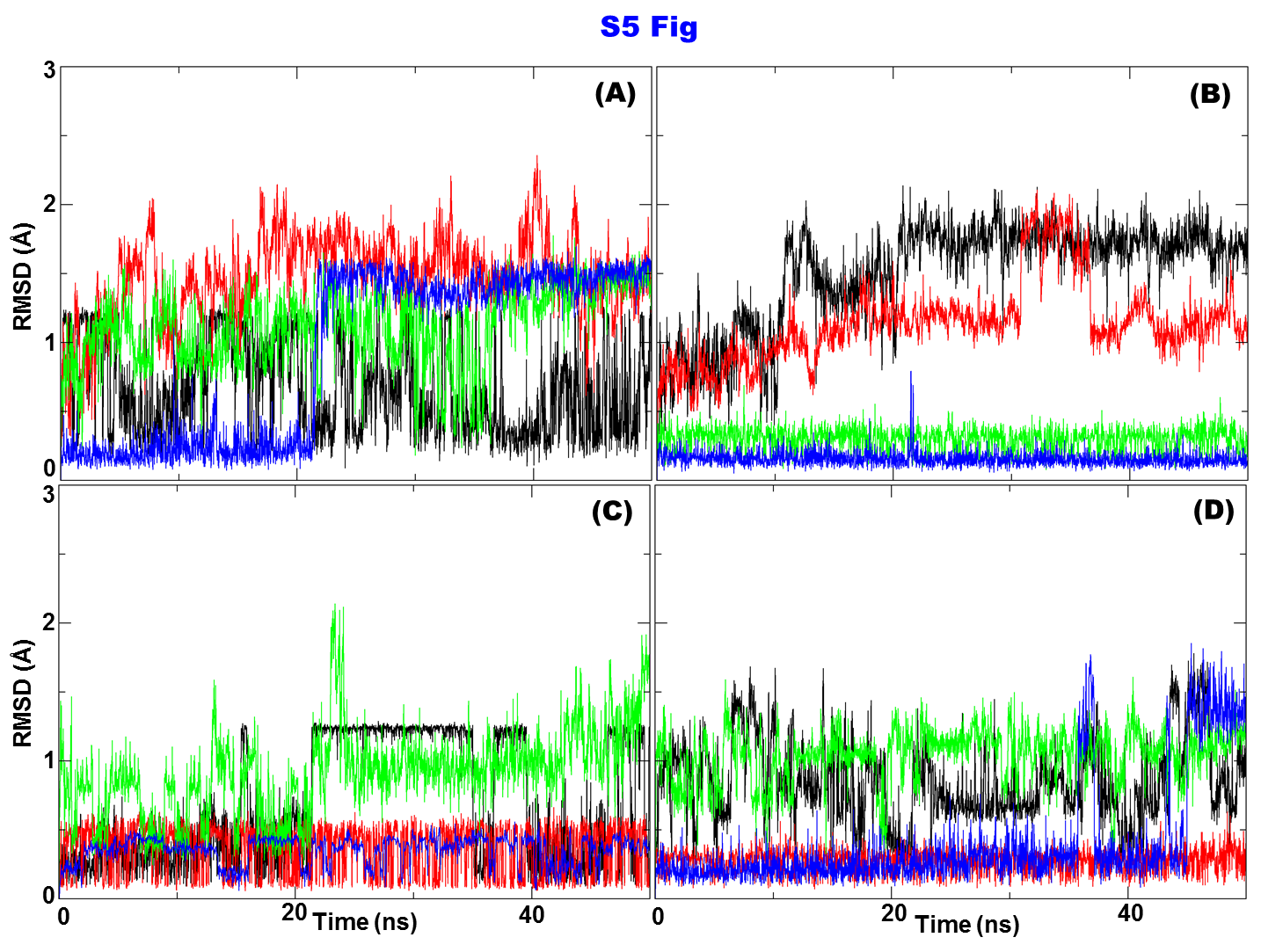


**S5 Fig**. **RMSD of bound ligand in protein-ligand complex**. RMSD of the backbone atoms of ligand substrates of rh-PON1-ligand complexes were plotted during the course of MDS to analyze the stability at the binding site. Panel (A-D) represent the ligand substrates, **(A)** - Pxn, **(B)** - Pha, **(C)** - *δ*-val, and **(D)** – TBBL. Proteins used were rh-PON1_(wt)_ (**—**), rh-PON1_(H115W,R192)_ (**—**), rh-PON1_(H115W,R192K)_ (**—**), and rh-PON1_(H115W,R192I)_ (**—**).
