# Supplementary material for: Toward Understanding the Catalytic Mechanism of Human Paraoxonase 1: Site-Specific Mutagenesis at Position 192
Source: PLoS One. 2016 Feb 1;11(2):e0147999. doi: 10.1371/journal.pone.0147999 (PMC4734699; doi:10.1371/journal.pone.0147999)
Supplement: S6 Fig — (DOCX) [file pone.0147999.s006.docx]

**Supporting information**


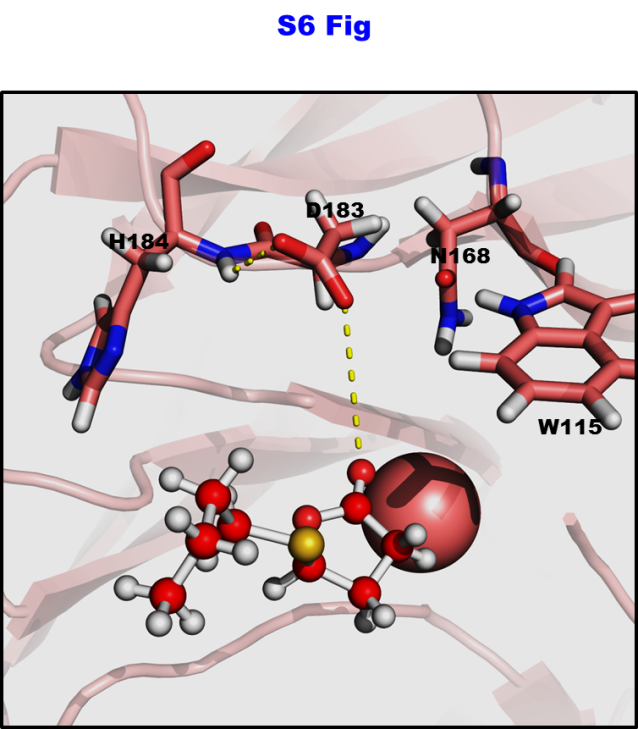


**S6 Fig.** **A snapshot showing binding conformation of TBBL in the active site of rh-PON1_(H115W,R192I)_**_._ Active site amino acids (D183, W115, N168 and H184) are shown in stick format and colored by atom type (red-oxygen, blue-nitrogen, salmon-carbon). TBBL is represented as stick model and colored by atom type (red-oxygen, yellow-sulphur, brick red-carbon). Catalytic calcium is shown as a sphere in salmon color. The yellow broken lines indicate the distance between D183 residue and the TBBL substrate. During the MD simulations, the oxygen atom of TBBL was found to be directed toward the carbonyl oxygen of D183 with an average distance ranging from 4.5 Å.
