# Supplementary material for: Toward Understanding the Catalytic Mechanism of Human Paraoxonase 1: Site-Specific Mutagenesis at Position 192
Source: PLoS One. 2016 Feb 1;11(2):e0147999. doi: 10.1371/journal.pone.0147999 (PMC4734699; doi:10.1371/journal.pone.0147999)
Supplement: S7 Fig — (DOCX) [file pone.0147999.s007.docx]

**Supporting information**


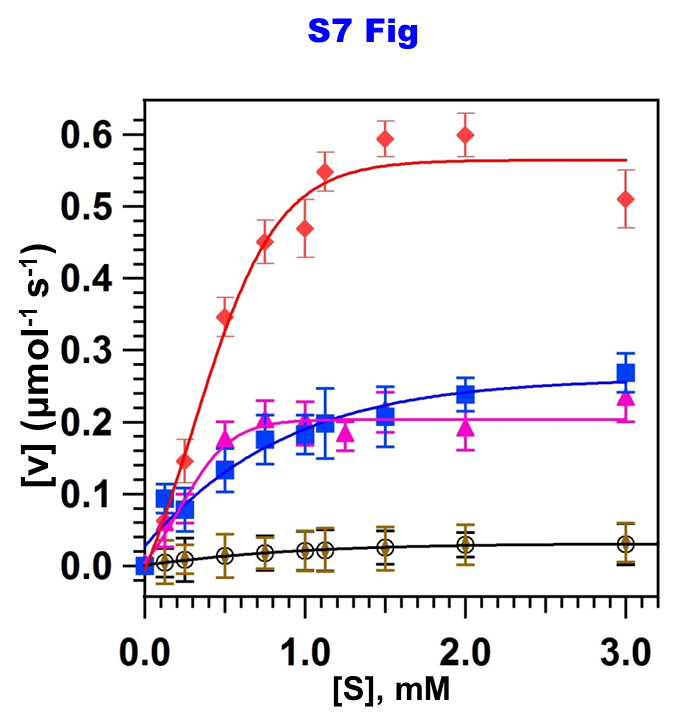


**S7 Fig. Michaelis-Menten plot for the hydrolysis paraoxon by the rh-PON1 enzymes**. The purified enzyme (0.4 μM final concentration) was mixed with different concentrations of Pxn (0-3 mM) and the hydrolytic activity was determined as described in the Materials and Methods. Initial velocities (v) of the Pxn hydrolysis and substrate concentrations (S) were used to generate the Michaelis-Menten plot for Pxn. The data were fitted by the Michaelis-Menten equation (R^2^ = 0.92-0.99) to obtain the kinetic parameters (presented in Table 1). Legends: (-🞅-), rh-PON1_(H115W,R192I)_; (-●-), rh-PON1_(H115W,R192S)_; (-■-); rh-PON1_(H115W,R192W)_; (-▲-); rh-PON1_(H115W,R192N)_ and (-◆-),rh-PON1_(H115W,R192A)_.
