# Supplementary material for: Toward Understanding the Catalytic Mechanism of Human Paraoxonase 1: Site-Specific Mutagenesis at Position 192
Source: PLoS One. 2016 Feb 1;11(2):e0147999. doi: 10.1371/journal.pone.0147999 (PMC4734699; doi:10.1371/journal.pone.0147999)
Supplement: S1 Table — (DOCX) [file pone.0147999.s008.docx]

**Supporting information**

| Primer | Oligonucleotide sequence |
| --- | --- |
| R192N | 5’-CTTCTTAGACCCGTATCTGAACAGCTGGGAGATGTACCTGG-3’  5’-CCAGGTACATCTCCCAGCTGTTCAGATACGGGTCTAAGAAG-3’ |
| R192D | 5’-CTTCTTAGACCCGTATCTGGATAGCTGGGAGATGTACCTG-3’  5’-CAGGTACATCTCCCAGCTATCCAGATACGGGTCTAAGAAG-3’ |
| R192E | 5’-CTTCTTAGACCCGTATCTGGAAAGCTGGGAGATGTACCTGG-3’  5’-CCAGGTACATCTCCCAGCTTTCCAGATACGGGTCTAAGAAG-3’ |
| R192S | 5’-CTTCTTAGACCCGTATCTGTCTAGCTGGGAGATGTACC-3’  5’-CAGGTACATCTCCCAGCTAGACAGATACGGGTCTAAGAAG-3’ |
| R192T | 5’-CTTCTTAGACCCGTATCTGACCAGCTGGGAGATGTACCTGG-3’  5’-CCAGGTACATCTCCCAGCTGGTCAGATACGGGTCTAAGAAG-3’ |
| R192W | 5’-CTTCTTAGACCCGTATCTGTGGAGCTGGGAGATGTACCTGG-3’  5’-CCAGGTACATCTCCCAGCTCCACAGATACGGGTCTAAGAAG-3’ |
| R192Y | 5’-CTTCTTAGACCCGTATCTGTATAGCTGGGAGATGTACCTG-3’  5’-CAGGTACATCTCCCAGCTATACAGATACGGGTCTAAGAAG -3’ |
| R192F | 5’-CTTCTTAGACCCGTATCTGTTTAGCTGGGAGATGTACCTG-3’  5’-CAGGTACATCTCCCAGCTAAACAGATACGGGTCTAAGAAG-3’ |
| R192L | 5’-CTTAGACCCGTATCTGCTGAGCTGGGAGATGTACCTG-3’  5’-CAGGTACATCTCCCAGCTCAGCAGATACGGGTCTAAG -3’ |
| R192I | 5’-CTTCTTAGACCCGTATCTGATTAGCTGGGAGATGTACCTG-3’  5’-CAGGTACATCTCCCAGCTAATCAGATACGGGTCTAAGAAG-3’ |
| R192V | 5’-CTTCTTAGACCCGTATCTGGTGAGCTGGGAGATGTACCTGG-3’  5’-CCAGGTACATCTCCCAGCTCACCAGATACGGGTCTAAGAAG-3’ |
| R192G | 5’-CTTCTTAGACCCGTATCTGGGTAGCTGGGAGATGTACCTG-3’  5’-CAGGTACATCTCCCAGCTACCCAGATACGGGTCTAAGAAG-3’ |
| R192A | 5’-CTTCTTAGACCCGTATCTGGCGAGCTGGGAGATGTACCTG-3’  5’-CAGGTACATCTCCCAGCTCGCCAGATACGGGTCTAAGAAG-3’ |
| R192P | 5’-CTTAGACCCGTATCTGCCGAGCTGGGAGATGTACCTG-3’  5’-CAGGTACATCTCCCAGCTCGGCAGATACGGGTCTAAG-3’ |
| R192H | 5’-CTTCTTAGACCCGTATCTGCATAGCTGGGAGATGTACCTGG 3‘  5’-CCAGGTACATCTCCCAGCTATGCAGATACGGGTCTAAGAAG 3' |
| R192M | 5’-CTTCTTAGACCCGTATCTGAGAGCTGGGAGATGTACC-3’  5’-GGTACATCTCCCAGCTCTCAGATACGGGTCTAAGAAG-3’ |

**Table S1**. **Primers used in the study**. Complementary oligonucleotides used in the study is generated using Primer X online software (www.bioinformatics/primerx/). Nucleotides for the desired mutation are underlined.
