# Supplementary material for: Toward Understanding the Catalytic Mechanism of Human Paraoxonase 1: Site-Specific Mutagenesis at Position 192
Source: PLoS One. 2016 Feb 1;11(2):e0147999. doi: 10.1371/journal.pone.0147999 (PMC4734699; doi:10.1371/journal.pone.0147999)
Supplement: S2 Table — (DOCX) [file pone.0147999.s009.docx]

**Supporting information**

FoldX analysis

| Amino acid alteration | ΔΔG (Kcal/mol) | Amino acid alteration | ΔΔG (Kcal/mol) |
| --- | --- | --- | --- |
| R192R | **0** | R192H | **11.48** |
| R192K | **-1.193** | R192F | **7.404** |
| R192Q | **-0.737** | R192L | **3.533** |
| R192N | **0.699** | R192I | **-1.789** |
| R192D | **1.555** | R192V | **-1.284** |
| R192E | **1.463** | R192M | **10.83** |
| R192S | **0.688** | R192P | **4.559** |
| R192T | **0.624** | R192G | **-0.041** |
| R192W | **3.119** | R192A | **-1.008** |
| R192Y | **4.869** |  |  |

**Table S2**: **Stability analysis of rh-PON1 mutants by FoldX algorithm**. The FoldX computed distribution of ΔΔG values of all possible amino acid substitutions at position 192 of rh-PON1 mutants. R192R is taken as a control.
