# Supplementary material for: Toward Understanding the Catalytic Mechanism of Human Paraoxonase 1: Site-Specific Mutagenesis at Position 192
Source: PLoS One. 2016 Feb 1;11(2):e0147999. doi: 10.1371/journal.pone.0147999 (PMC4734699; doi:10.1371/journal.pone.0147999)
Supplement: S1 Text — (DOCX) [file pone.0147999.s010.docx]

**Supporting information**

**Toward understanding the catalytic mechanism of human paraoxonase 1: site-specific mutagenesis at position 192**

**Geetika Aggarwal^1,^ Rameshwar Prajapati^2^, Rajan K. Tripathy^1^, Priyanka Bajaj^1^, A.R. Satvik Iyengar^1^, Abhay T. Sangamwar^2^ and Abhay H. Pande^1,~~3*~~^**

^1^Department of Biotechnology, National Institute of Pharmaceutical Education and Research (NIPER), Sector 67, S.A.S. Nagar (Mohali) -160062, Punjab, India.

^2^Department of Pharmacoinformatics, National Institute of Pharmaceutical Education and Research (NIPER), Sector 67, S.A.S. Nagar (Mohali) -160062, Punjab, India.

**^~~3*~~^To whom correspondence should be addressed**: Abhay H. Pande, Department of Biotechnology, National Institute of Pharmaceutical Education and Research (NIPER), Sector 67, S.A.S. Nagar (Mohali) -160 062, Punjab, India. Tel.: +91 172 2214 682, Fax: +91 172 2214 692. E-mail: [apande@niper.ac.in](mailto:apande@niper.ac.in)

**Supporting Methods**

**Sequence alignment**

Blastp search was performed using h-PON1 amino acid sequence as a query sequence against non-redundant protein sequence database. Mammalian PON1 sequences were selected on the basis of identical conserved residues of PON family (E53, D54, N168, D269, N224, 268, D269). These sequences have sequence identity varying from 80-99%. Sequence alignment was performed using free online software-clustalW (www.ebi.ac.uk/Tools/msa/clustalow). Conserved residues (E53, D54, H115, H134, N168, D183 and H184) and position 192 are highlighted manually with the Jalview software. Sequences, despite of their low sequence identity (28-35%) but containing all the conserved residues of the PON family, were also selected from NCBI database. They were also aligned against the h-PON1 sequence using clustalW.

**FoldX analysis**

The effect on the structural stability of rh-PON1 mutants due to mutations at position 192 was analyzed by FoldX algorithm using the FoldX web server (http://foldx.crg.es/) [1-3]. By using this algorithm, distribution of ΔΔG values of all possible amino acid substitutions at position 192 of rh-PON1 mutants was calculated.

**Supporting references**

1. Tokuriki N, Stricher F, Serrano L, Tawfik DS (2008) How protein stability and new functions trade off. PLoS ONE 4: 1-7.
2. Simoes-Correia J, Figueiredo J, Lopes R, Stricher F, Oliveira C, Serrano L, et al (2012) E-Cadherin destabilization accounts for the pathogenicity of missense mutations in hereditary diffuse gastric cancer. PLoS ONE 7: 1-11.
3. Tokuriki N, Stricher F, SchymkowitzJ, Serrano L, Tawfik DS (2007) The stability effects of protein mutations appear to be universally distributed. J Mol Biol 369, 1318–1332.
